# SF3A2: a promising therapeutic target and predictive biomarker for immunotherapy in colorectal cancer

**DOI:** 10.7150/ijms.123448

**Published:** 2026-01-01

**Authors:** Changjiang Yang, Shidong Zhao, Long Zhao, Yuanpei Lin, Yingjiang Ye, Caihong Wang, Zhanlong Shen

**Affiliations:** Department of Gastroenterological Surgery, Peking University People's Hospital, No.11 Xizhimen South Street, Beijing, 100044, PR China.

**Keywords:** colorectal cancer, SF3A2, prognosis, immune microenvironment, immunotherapy

## Abstract

Colorectal cancer (CRC) is one of the most prevalent malignancies globally and poses a substantial threat to human health. The current understanding of the biological significance of Splicing Factor 3a Subunit 2(SF3A2) in CRC remains limited. In this study, the upregulation of SF3A2 in CRC was identified through TMT-based quantitative proteomic screening of surgically resected paired primary cancer and normal epithelial tissues. Patients with elevated SF3A2 expression exhibited reduced survival compared to those with lower expression levels. Knockdown of SF3A2 significantly decreased cell proliferation, migration, and invasive properties both in vivo and in vitro. Bioinformatics enrichment analysis revealed that SF3A2 was involved in alternative splicing and functioned as an oncogene by modulating the expression of genes associated with critical tumorigenesis pathways and functions. Furthermore, immune infiltration analysis using multiple algorithms (including TIMER, EPIC, QUANTISEQ, and MCPCOUNTER) indicated an inverse relationship between SF3A2 expression levels and the presence of various immune cell types. Concurrently, predictions derived from the TIDE algorithm corroborated that patients exhibiting elevated SF3A2 expression were likely to experience a diminished response to immunotherapy. In summary, SF3A2 emerged as a promising therapeutic target for CRC and served as a novel biomarker for forecasting responses to immunotherapeutic interventions.

## Background

Colorectal cancer (CRC) persists as a principal contributor to cancer-associated deaths globally^1^. Its intricate nature stems from multiple signal transduction pathways that regulate cellular growth, viability, differentiation, and programmed cell death. These interconnected signaling networks shape not only malignant characteristics but also immune responses and the tumor microenvironment (TME), ultimately affecting treatment outcomes^2^. Consequently, identifying potential CRC drivers and elucidating their mechanisms remains crucial for discovering additional effective therapeutic targets against CRC.

While the advent of immune checkpoint blockade (ICB) therapy, targeting pathways such as PD-1/PD-L1 and CTLA-4, has revolutionized the treatment landscape for multiple malignancies, its efficacy in CRC is strikingly heterogeneous and largely confined to the small subset of patients with microsatellite instability-high (MSI-H) or mismatch repair-deficient (dMMR) tumors. The majority of CRC patients, characterized by microsatellite stable (MSS) or proficient mismatch repair (pMMR) status, exhibit primary resistance to ICB monotherapy, underscoring the critical need to identify novel molecular determinants underlying the immunosuppressive tumor microenvironment (TME) and intrinsic immune evasion mechanisms in this prevalent subgroup^3^, ^4^.

Alternative splicing (AS) functions as an essential regulatory mechanism at the transcriptional level, generating transcript variety and affecting protein structure and function. Dysregulation of AS has been associated with numerous pathological conditions, especially malignancies. Such disruptions can modify gene splicing patterns, resulting in altered or compromised protein functions^5^. Accumulating evidence has revealed that defects in alternative splicing contribute to a variety of features of cancer development, including the modulation of cancer heterogeneity, evasion of apoptosis of cancer cells, rewiring cancer metabolism, and facilitating cancer metastasis via the epithelial to mesenchymal transition process^6^. Furthermore, investigations demonstrate that alternative splicing influences the tumor microenvironment by modulating tissue remodeling, immune responses, and inflammatory processes.

Among these, splicing factors exhibit a fundamental function in alternative splicing regulation. The impairment of splicing elements represents a primary factor contributing to numerous irregular AS occurrences in human malignancies^7^. Examining the splicing elements participating in this irregular splicing mechanism could offer novel therapeutic insights to comprehend CRC's malignant characteristics^8^. Splicing factor 3a subunit 2 (SF3A2) functions as a constituent of the splicing element SF3A complex, facilitating 17S U2 snRNP. Current research indicates SF3A2's immediate involvement in mitotic chromosome distribution^9^. In addition, SF3A2 had a pivotal role in the pathophysiology of myocardial ischemia reperfusion injury. On the one hand, SF3A2 was involved in NEDD4-mediated macrophage pyroptosis in myocardial ischemia reperfusion^10^; on the other hand, the inhibition of SF3A2 acetylation at lysine 10 could promote Fscn1 alternative splicing against myocardial ischemic/reperfusion injury^11^. Recent investigations have increasingly focused on its irregular expression's impact on tumor formation and progression. Investigations demonstrate the PQBP1-SF3A2-UBA52-PCK2 pathway's connection to osteosarcoma recurrence and metastasis^12^. SF3A2 was a valuable prognostic indicator and potential therapeutic target for enhanced HNSCC treatment outcomes^13^. Additionally, SF3A2 enhances progression and cisplatin resistance in triple-negative breast cancer through MKRN1 alternative splicing^14^. Despite these significant findings, SF3A2's biological roles and underlying molecular mechanisms in CRC remain largely unexplored.

Our proteomic investigation contrasted protein abundance patterns between malignant colorectal specimens and neighboring healthy tissues. The analysis revealed numerous differentially abundant proteins, with splicing regulator SF3A2 exhibiting notable elevation in CRC samples. We comprehensively examined SF3A2 to elucidate its biological roles and underlying mechanisms. Multiple public datasets and patient specimens demonstrated that elevated SF3A2 levels influenced CRC advancement and clinical outcomes. Experimental reduction of SF3A2 demonstrated tumor-inhibiting properties in biological systems and laboratory conditions. Moreover, our investigation into the relationship between SF3A2 and the immune landscape of CRC revealed negative correlations between SF3A2 expression levels and various immune cell types. The tumor immune microenvironment in patients exhibiting high SF3A2 expression was notably deficient in most immune cells, indicative of an immune desert microenvironment, often referred to as a "cold tumor." Notably, CRC patients exhibiting elevated SF3A2 expression demonstrated a suboptimal response to anti-tumor immunotherapy. Collectively, these findings shed light on previously unexplored aspects of SF3A2's role in CRC, suggesting its potential as a target for therapeutic intervention and as a novel biomarker for predicting responses to immunotherapy in CRC patients.

## Materials and Methods

### Patient and clinical specimens

The clinical tissues used in this study were obtained from surgical resections performed at Peking University People's Hospital (Beijing, China) with informed consent and institutional ethical approval. Patient selection was conducted based on the following criteria. For inclusion, patients were required to have: (1) a histologically confirmed diagnosis of primary colorectal adenocarcinoma; (2) undergone curative surgical resection with available high-quality paired tumor and normal tissue specimens; and (3) complete clinicopathological data. Key exclusion criteria encompassed: (1) a history of any other malignant tumor; (2) receipt of neoadjuvant chemotherapy or radiotherapy prior to surgery; (3) incomplete medical records. All surgical patients provided both tumor and normal tissue samples. Normal tissue specimens were harvested at a minimum distance of 5 cm from the neoplastic site. Each tumor specimen had a corresponding para-tumor sample. Rapid frozen section pathology confirmed specimen validity by a pathologist. The specimens for immunohistochemistry (IHC) analysis were constructed using formalin-fixed, paraffin-embedded (FFPE) tissue microarrays. The detailed clinical and pathological characteristics of the patients were provided in supplementary Table S1.

### Tandem mass tag (TMT)-based proteomic profiling analysis

In this study, we established an integrated quantitative proteomics workflow by combining TMT labeling, high-pH HPLC fractionation, and liquid chromatography-tandem mass spectrometry (LC-MS/MS). Jingjie PTM Biolab Co., Ltd. (Hangzhou, China) executed the comprehensive proteomic analysis.

### Cell culture, transfection, and generation of stable cell lines

Human CRC cell lines LOVO and HCT116 were procured from the Cell Bank of the Chinese Academy of Science (Shanghai, China). LOVO was maintained in F12K medium (Gibco). HCT116 was kept in DMEM medium (Gibco). All culture media were enriched with 10% fetal bovine serum (FBS; Gibco) and 1% penicillin and streptomycin (PS; Gibco). The cells were cultivated at 37°C in a humidified chamber with 5% CO_2_. For lentiviral transduction, cells were seeded in 6-well plates and infected with lentiviral particles at a multiplicity of infection (MOI) of 20 in the presence of 6 µg/mL polybrene. After 24 hours of incubation, the virus-containing medium was replaced with fresh complete medium. Stable cell lines were selected using 2 µg/mL puromycin for at least one week.

### Data acquisition and preprocessing

Gene expression profiles and clinical information from tumor and normal samples were obtained through the TCGA portal (https://portal.gdc.cancer.gov/). The investigation incorporated datasets GSE89076, GSE110223, GSE110224, and GSE113513, which were accessed from GEO (http://www.ncbi.nlm.nih.gov/geo).

### Immunohistochemistry (IHC) Staining

Tissue specimens embedded in paraffin underwent deparaffinization, hydration restoration, and quenching of endogenous peroxidase activity. Following PBS solution rinses (pH 7.4), the specimens received treatment with 10% caprine serum before exposure to SF3A2 primary antibody (1:200 dilution, 15596-1-AP, Proteintech, China) at 4°C overnight, succeeded by secondary antibody application for 60 minutes at ambient temperature following manufacturer protocols. Visualization occurred through diaminobenzidine reaction, followed by hematoxylin counterstaining. SF3A2 expression underwent evaluation by two independent pathologists, considering both cellular positivity distribution and staining intensity. The positive cell distribution received classification as follows: 0 (0-10%), 1 (10-40%), 2 (40-70%), and 3 (>70%), while staining intensity was categorized as 0 (mild), 1 (intermediate), and 2 (pronounced). The final IHC score resulted from combining these parameters. SF3A2 expression levels were ultimately classified as: minimal (0-2 points) or substantial (3-5 points).

### Lentivirus and viral infections

The lentiviral constructs containing shSF3A2 were procured from GeneChem (Shanghai, China). Control experiments utilized GFP-expressing empty vector lentiviruses as negative controls. Cellular infection proceeded using lentiviral particles supplemented with polybrene, followed by puromycin selection. Following the transfection process, cellular material was collected, and RT-qPCR/western blot analysis confirmed transfection efficacy.

### Western blotting

Total protein was procured from the specimens utilizing extraction buffer (P0013; Beyotime BioTech Inst) containing protease and phosphatase inhibitors. Identical quantities of protein underwent separation via 10% SDS-PAGE and were transferred to polyvinylidene difluoride membranes (Millipore). The membranes underwent blocking with 5% non-fat milk for 1 h at ambient temperature, followed by overnight incubation with anti-SF3A2 (1:1000, 15596-1-AP, Proteintech) and β-Actin (1:1000, 66009-1-Ig; Proteintech) antibodies at 4°C. The membranes subsequently underwent incubation with secondary antibodies, and protein signals were detected through enhanced chemiluminescence detection using a Bio-Rad gel imaging system (Bio-Rad). For quantitative analysis, the band intensities of SF3A2 and β-Actin were measured using ImageJ software. The relative expression level of SF3A2 in each sample was calculated as the ratio of the SF3A2 band intensity to the corresponding β-Actin band intensity.

### Quantitative Real-time PCR (RT-qPCR)

Total RNA was isolated via the Trizol method and subsequently reverse transcribed using the FastKing-RT SuperMix kit (TIANGEN, Cat. No. KR118). The RT-qPCR was caried out utilizing the iTaq Universal SYBR® Green SuperMix (2×) system (Bio-Rad, Cat.No.1725122). Target gene mRNA expression levels were determined using the 2^-ΔΔCt^ method. The primer sequences are as followed:

SF3A2-F: 5'-GATTGACTACCCTGAGATCGCC-3'

SF3A2-R: 5'-CTCCCGGTTCCAGTGTGTC-3'

β-Actin-F: 5'-CATGTACGTTGCTATCCAGGC-3'

β-Actin-R: 5'-CTCCTTAATGTCACGCACGAT-3'

Cell proliferation assay and colony formation assays

Cell growth was evaluated using the Cell Counting Kit-8 (CCK-8) methodology. For the CCK-8 (Dojindo) procedure, 1000 cells per well were placed in 96-well plates containing 100ul growth medium. 10μl CCK-8 reagent was introduced at designated intervals. Following cultivation at 37°C in 5% CO_2_ conditions, the reaction outcomes were measured spectrophotometrically at 450 nm using a plate analyzer. Each test was conducted three times. For colony establishment tests, 500 cells were distributed in triplicate across 6-well plates. Following approximately 2 weeks of cultivation, surviving colonies underwent methanol fixation and 0.1% crystal violet staining.

### Transwell assay

Cell migration and invasion analyses were conducted using a 24-well plate equipped with an 8.0μm pore polycarbonate barrier (Corning Costar, Corning), either without or with Matrigel coating. The bottom compartment contained 750μl DMEM enriched with 20% FBS. CRC cells (5×105 cells/well) were dispersed in 200μl serum-free medium and introduced to the upper compartment. Following 36-48 h incubation, migrated CRC cells underwent fixation and 0.1% crystal violet staining before counting. Cell quantities were determined by examining three arbitrary fields under microscopy, with the mean value used for numerical assessment.

### Mouse xenograft tumor model

All animal experimental protocols were approved by the Ethics Committee of Peking University People's Hospital. BALB/c Nude mice at six weeks of age were obtained from Animal Laboratory of Peking University People's Hospital (Beijing, China). Each animal underwent an examination to confirm health status and absence of disease prior to tumor implementation. HCT116 cells numbering 2 × 10^6^ were administered subcutaneously into nude mice. At the end of the experiment, the mice were euthanized in accordance with the approved animal protocol, and the tumors were harvested, and their volumes and weights were documented. The Ki67 polyclonal antibody (proteintech, 27309-1-AP,1:10000) was used for IHC. For the quantitative analysis of Ki67 staining in xenograft tumor tissues, whole slide images were analyzed using ImageJ software. The Ki-67 positive area (DAB-stained) and the total tumor area were measured. The proliferation index for each sample was calculated as the percentage of the Ki-67 positive area relative to the total tumor area.

### Enrichment analysis

The R software package ClusterProfiler (3.6.3) was utilized to perform gene set enrichment analysis (GSEA) alongside Gene Ontology (GO) and Kyoto Encyclopedia of Genes and Genomes (KEGG) examinations. The GO investigation encompassed cellular components (CC), molecular functions (MF), and biological processes (BP). GSEA functions as a computational method to evaluate the statistical significance and consistency of differences between two biological conditions based on predetermined gene collections. The adjusted *P*-value and normalized enrichment score (NES) were employed to classify enriched pathways within each phenotype. Gene sets were markedly enriched when meeting adjusted p<0.05 thresholds and false discovery rate (FDR) < 0.25.

### Immune infiltration analysis

The ESTIMATE^15^ algorithm was employed to calculate the ImmuneScore, StromalScore and ESTIMATEScore for each tumor sample. Furthermore, the TIMER^16^, EPIC^17^, QUANTISEQ^18^, and MCPCOUNTER^19^ methodologies were applied to evaluate the association between SF3A2 expression and infiltrating immune cells. The Tumor Immune Dysfunction and Exclusion (TIDE)^20^ platform was utilized to assess responses to immunotherapy. The relationship between SF3A2 expression and these immune indices was analyzed using Spearman's correlation analysis. To compare the levels of immune cell infiltration between low and high SF3A2 expression groups, the Wilcoxon rank-sum test was conducted.

### Statistical analysis

All statistical analyses were performed using R software (version 4.2.1).For data that conformed to a normal distribution, a parameter test was used, and for data that did not conform to a normal distribution, a non-parametric test was used. Patient survival evaluations were conducted employing KM methodology and log-rank statistical testing. The correlation between clinical-pathological parameters and patient outcomes underwent assessment via univariate and multivariate Cox proportional hazards model. Predictive factors yielding *P*-values below 0.1 in the univariate evaluation were selected for subsequent multivariate examination. Following this, multivariate Cox proportional hazards model was utilized to identify independent prognostic markers. For high-throughput analyses, such as gene set enrichment analysis (GSEA), the false discovery rate (FDR) was controlled using the Benjamini-Hochberg method.

## Results

### Differential expression of proteins in tumor and normal tissues of CRC

**Figure 1:**
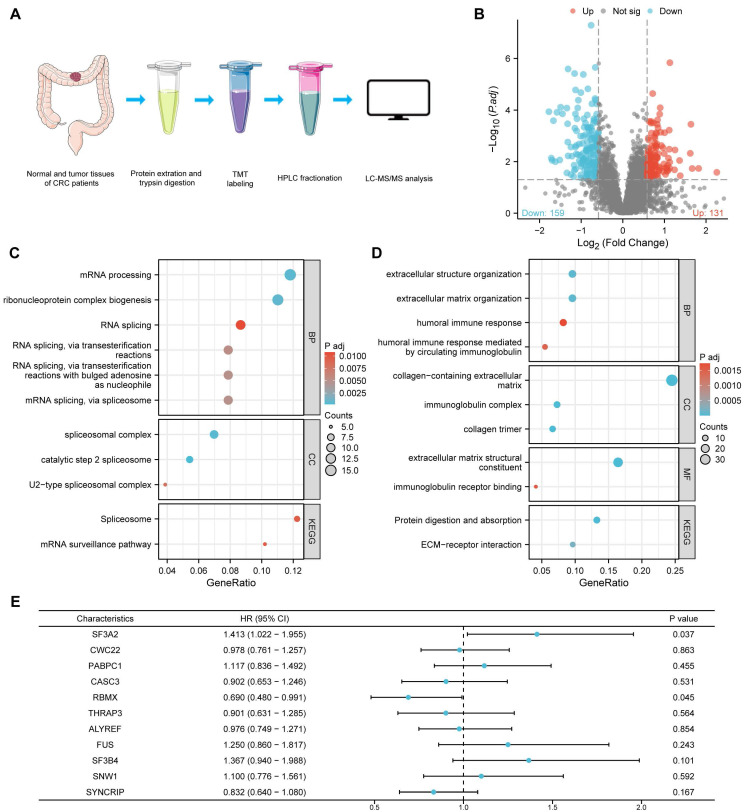
Differential expression of proteins in tumor and normal tissues of CRC based on proteomics. A. A schematic diagram describing the screening of dysregulated proteins in the quantitative proteome dataset. B. Volcano plot of differentially expressed proteins between CRC tissues and matched adjacent normal tissues. C. Gene ontology (GO) analysis and KEGG pathway analysis of up-regulated proteins. D. Gene ontology (GO) analysis and KEGG pathway analysis of down-regulated proteins. E. Forest plot of hazard ratios demonstrating the prognostic values of AS-related genes based on univariate Cox proportional hazards model (high risk genes: HR>1, low risk genes: HR<1).

To examine protein expression patterns in CRC, we gathered 9 matched sets of CRC specimens and neighboring healthy tissue samples for comparative protein analysis via proteomics. Setting the differential expression threshold at fold change >1.5 and statistical significance at *P* value < 0.05, our analysis revealed 131 proteins showing elevated expression and 159 proteins exhibiting reduced expression within the cancer specimens among the measured proteins (Figure. 1A, B, Supplementary Table S2).

In order to comprehensively explore the biological roles of the differentially expressed proteins, these up-regulated and downregulated proteins were submitted to GO and KEGG pathway enrichment evaluation. The GO analysis results encompassed three categories: biological process (BP), cellular component (CC), and molecular function (MF). In this investigation, proteins with enhanced expression were primarily concentrated in mRNA processing (BP), ribonucleoprotein complex biogenesis (BP), RNA splicing (BP), spliceosomal complex (CC) and splicosome (KEGG) (Figure 1C) while downregulated proteins were associated with extracellular structure organization (BP), extracellular matrix organization (BP), collagen-containing extracellular matrix (CC), extracellular matrix structural constituent (MF) and protein digestion and absorption (KEGG) (Figure 1D).

The GO and KEGG pathway assessment findings suggested that the dysregulations of RNA splicing were important driving forces for the carcinogenesis of CRC. To identify the most prognostically relevant splicing factor among the dysregulated candidates, we selected the 11 aberrantly expressed proteins involved in RNA splicing (SF3A2, CWC22, PABPC1, CASC3, RBMX, THRAP3, ALYREF, FUS, SF3B4, SNW1, and SYNCRIP) for further investigation. A univariate Cox proportional hazards model for overall survival (OS) was performed to compare their prognostic power. Among these candidates, SF3A2 exhibited the highest hazard ratio (HR = 1.413) and the smallest *P*-value (p = 0.037) for predicting poor OS (Figure 1E), establishing it as the most robust prognostic indicator. This comparison provided the primary rationale for our subsequent focus on SF3A2.

### SF3A2 was aberrantly up-regulated in CRC tissues

**Figure 2:**
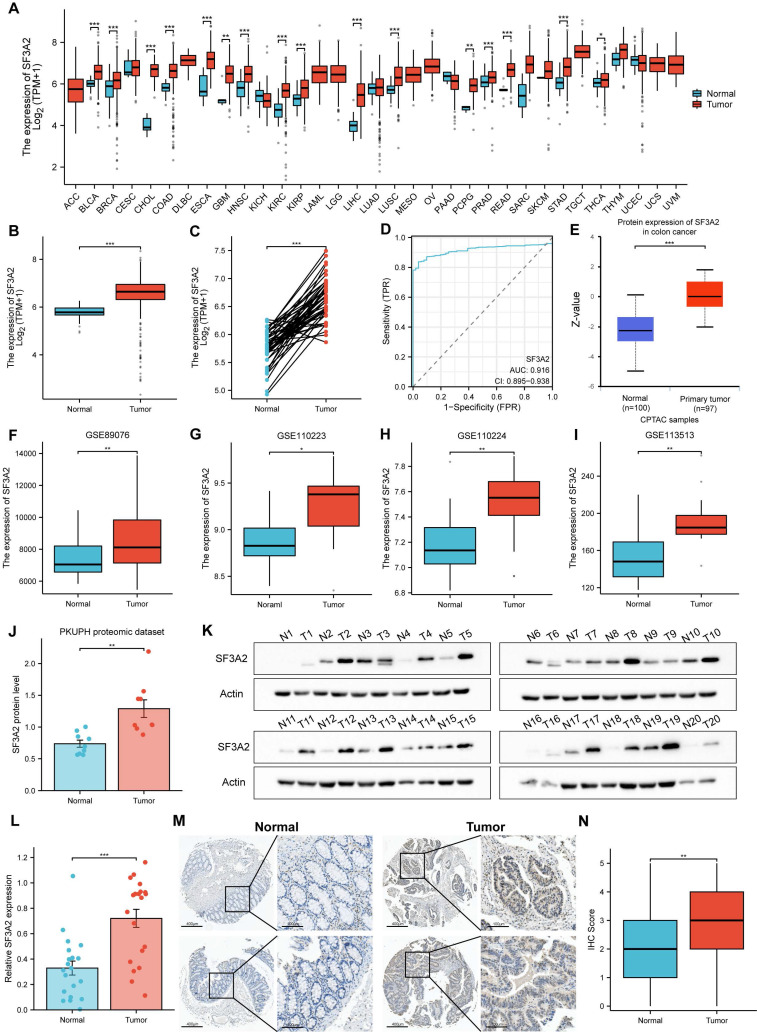
SF3A2 was aberrantly up-regulated in CRC tissues. A. SF3A2 mRNA expression in pan-cancer tissues compared with normal tissues based on TCGA. B and C. SF3A2 mRNA expression was upregulated in CRC compared with normal tissues based on TCGA. D.ROC analysis of SF3A2 in the diagnosis of CRC. E. SF3A2 protein expression was upregulated in CRC compared with normal tissues based on CPTAC. F-I. SF3A2 mRNA expression was upregulated in CRC compared with normal tissues based on GEO. J. The protein level of SF3A2 in the PKUPH quantitative proteome dataset. K and L. Western blotting to detect SF3A2 expression in 20 paired normal and CRC tissues. M and N IHC staining showing SF3A2 protein expression was upregulated in CRC samples compared with normal tissues. (ns represents no significance, * p < 0.05, ** p < 0.01, *** p < 0.001).

Utilizing TCGA database resources, we investigated SF3A2 gene expression across diverse human cancer types versus normal specimens. SF3A2 mRNA levels exhibited significant elevation in malignancies compared to corresponding normal tissue samples (Figure 2A). Notably, SF3A2 demonstrated marked upregulation in CRC (Figure 2B, C). ROC curve analysis yielded an AUC of 0.916 (95% CI = 0.895-0.938), suggesting strong discriminatory capability between tumor and normal tissues (Figure 2D). Additional validation of SF3A2's elevated expression in CRC came from CPTAC and GEO datasets (GSE89076, GSE110223, GSE110224, and GSE113513) (Figure 2E-I). Within PKUPH proteomic cohorts, SF3A2 expression was significantly elevated in CRC specimens compared to normal tissue (Figure 2J). For additional verification, we assessed SF3A2 protein levels across 20 paired CRC and normal tissue samples via immunoblotting. The detailed clinical and pathological characteristics of the patients included in the WB study are provided in supplementary Table S1. Consistent with previous findings, SF3A2 expression demonstrated significant elevation in CRC specimens compared to normal counterparts (Figure 2K, L). To evaluate SF3A2's tissue distribution in human CRC specimens, we constructed tissue microarrays encompassing 75 CRC samples. The detailed clinical and pathological characteristics of the patients included in the IHC study are provided in supplementary Table S1. Immunostaining with validated SF3A2 antibodies demonstrated increased SF3A2 levels in tumor tissue relative to normal tissue (Figure 2M, N). These observations collectively indicate SF3A2's elevated expression in colorectal cancer tissues and its potential role as a CRC driver, warranting further investigation into its function in CRC development.

### High SF3A2 expression predicted poor prognosis in CRC patients

**Figure 3:**
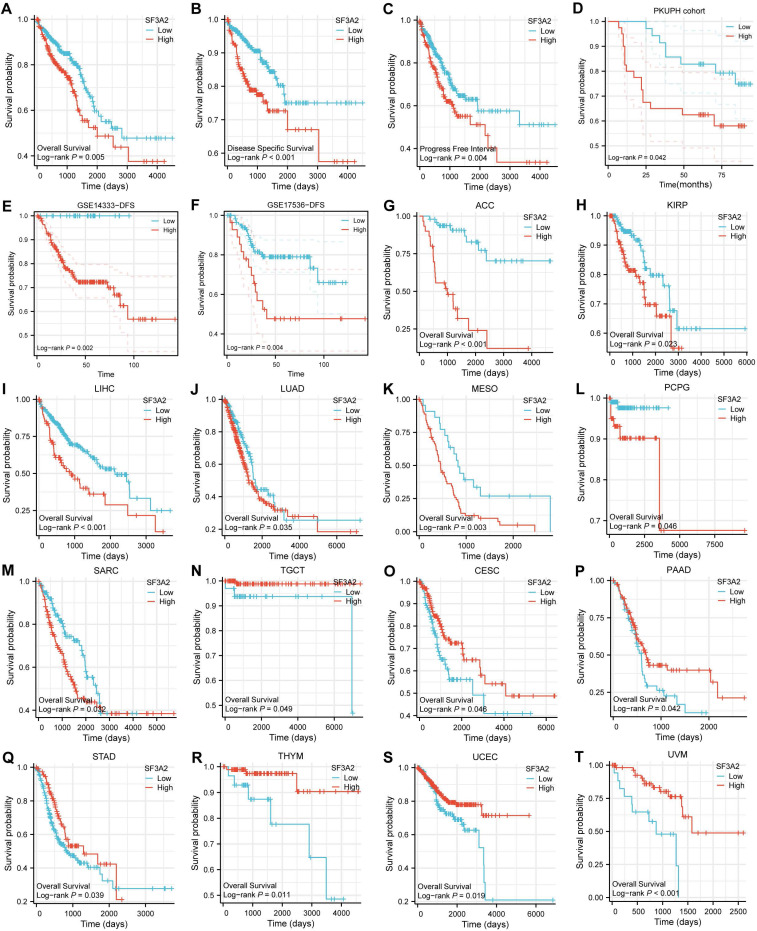
Associations between SF3A2 expression and its prognostic significance in CRC based on TCGA database A-C. The Kaplan-Meier curves show that CRC patients with a higher expression of SF3A2 had a shorter OS time, DSS time, and PFI time. D. Kaplan-Meier plot for SF3A2 expression to evaluate the probability of OS based on IHC cohort. E and F. Kaplan-Meier plot for SF3A2 expression to evaluate the probability of DFS based on GEO cohort. G to T. The examination of SF3A2's pan-cancer prognostic implications through Kaplan-Meier survival assessment. (ns represents no significance, * p < 0.05, ** p < 0.01, *** p < 0.001).

We evaluated SF3A2's prognostic significance in predicting overall survival (OS), disease-specific survival (DSS), and progression-free interval (PFI) outcomes across CRC cases. Kaplan-Meier survival assessment using TCGA data demonstrated that patients who expressed lower SF3A2 levels had enhanced OS, DSS, and PFI. (Figure 3A-C). Through univariate and multivariate examinations, elevated SF3A2 emerged as an autonomous risk indicator for CRC patients regarding OS (Table 1) and DSS (Table 2), rather than PFI (Table 3). In the IHC cohort, high levels of analysis confirmed that elevated SF3A2 were also significantly correlated markedly with reduced OS duration in CRC patients (Figure 3D). External validation via GEO datasets (GSE14333, GSE17536) showed negative associations between heightened SF3A2 levels and DFS (Figure 3E, F). These findings suggest that enhanced SF3A2 expression indicates poorer CRC patient outcomes. Furthermore, examining SF3A2's pan-cancer prognostic implications through Kaplan-Meier survival assessment revealed that increased SF3A2 corresponded to diminished OS in ACC, KIRP, LIHC, LUAD, MESO, PCPG, SARC patients (Figure 3G-M). Interestingly, and highlighting the context-dependent role of SF3A2, elevated SF3A2 expression was associated with extended OS in several other cancer types, including TGCT, CESC, PAAD, STAD, THYM, UCEC, and UVM patients (Figure 3N-T). This suggested that the biological function of SF3A2 might vary across different cellular contexts, a phenomenon worthy of future investigation. Nevertheless, its role in CRC is consistently associated with poor prognosis.

### SF3A2 accelerated cell proliferation, colony formation, migration, and invasion of CRC

**Figure 4:**
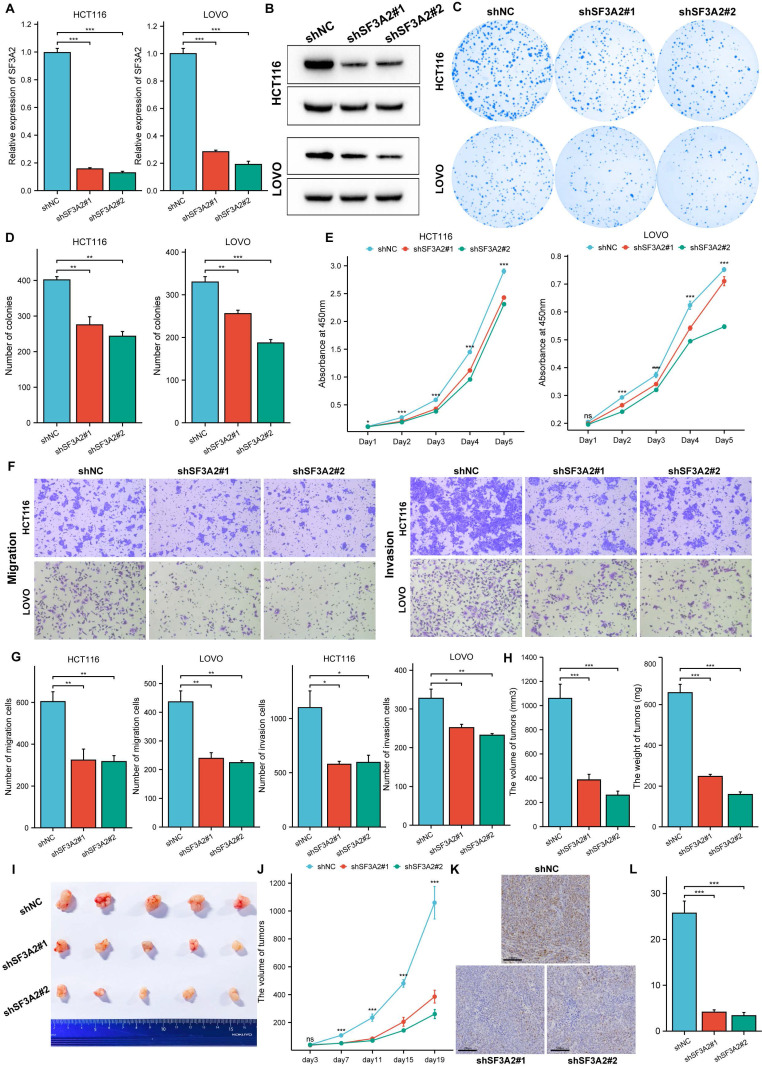
Knockdown of SF3A2 impaired cell proliferation, colony formation, migration, and invasion of CRC. A and B. RT-qPCR and Western blotting analyses of SF3A2 protein levels in HCT116 and LOVO cells stably expressing shNC or shSF3A2 (#1 and #2). C and D. Colony formation of HCT116 and LOVO (control or SF3A2 knockdown). The number of colonies formed was counted by ImageJ(n=3). E. CCK-8 assay evaluated the proliferation of SF3A2 knockdown HCT116 and LOVO cells in vitro(n=5). F and G. Transwell assays to evaluate the ability of migration and invasion of HCT116 and LOVO (control or SF3A2 knockdown). The number of cells were counted by ImageJ(n=3). H to J Control HCT116 cells or SF3A2-knockdown HCT116 cells were implanted in BALB/c Nude mice. Tumors were resected and measured 19 days later. K. Ki67 staining of tumor tissue from the control or SF3A2-knockdown group. L. Quantitative analysis of the Ki67 proliferation index (percentage of positive area). (ns represents no significance, * p < 0.05, ** p < 0.01, *** p < 0.001).

To investigate SF3A2's biological role in CRC, we reduced endogenous SF3A2 in HCT116 and LOVO cells through lentiviral vector introduction expressing two distinct shRNAs (shSF3A2 #1 and #2). We confirmed shSF3A2 lentivirus effectiveness through RT-qPCR and western blot analyses (Figure 4A, B). SF3A2 suppression diminished cellular proliferation compared to controls, as demonstrated by colony formation (Figure 4C, D) and CCK-8 (Figure 4E) evaluations. Additionally, transwell experiments indicated that SF3A2 reduction markedly decreased cellular migration and invasion capabilities (Figure 4F, G). To evaluate SF3A2's impact on colon cancer cells within living organisms, we developed tumor xenograft models. Following the implantation of SF3A2-depleted HCT116 cells (n = 5), we observed decreased tumor growth rates and diminished tumor dimensions and mass. Furthermore, immunohistochemical staining for Ki67 revealed a marked reduction in proliferating cells in the shSF3A2 group compared to the shNC controls (Figure 4H-L).

### The potential biological mechanism of SF3A2 involved in the malignant progression of CRC

**Figure 5:**
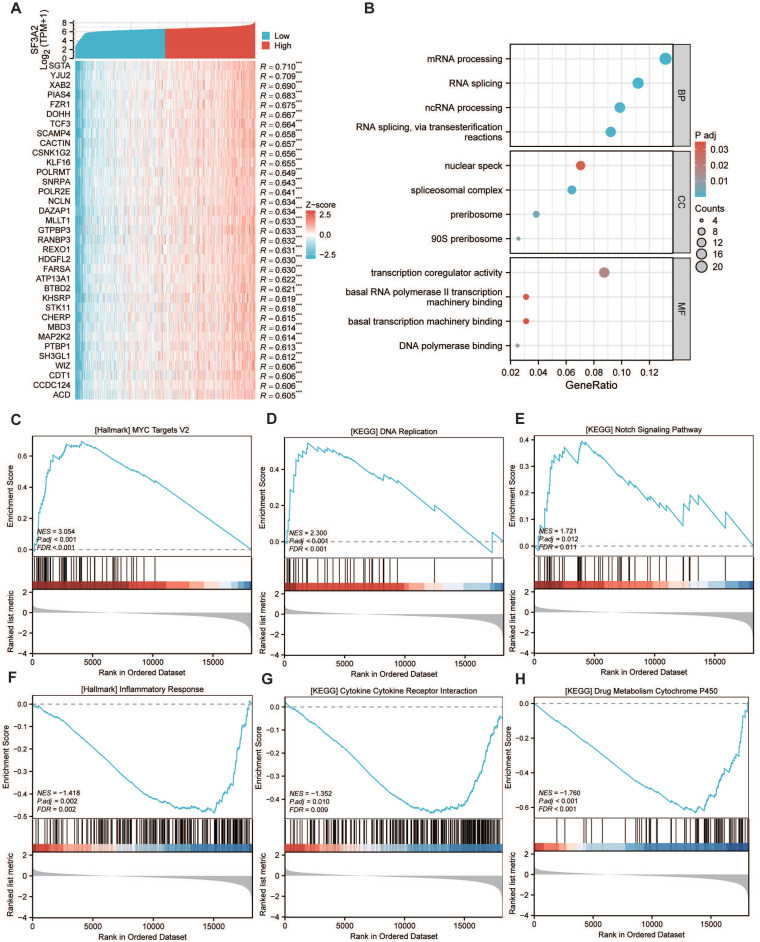
The potential biological mechanism of SF3A2 involved in the malignant progression of CRC.A. Heatmap illustrating the top 35 genes positively correlated with SF3A2 in CRC. Red represents high expression, and blue represents low expression. B. GO and KEGG pathway analysis of SF3A2 co-expressed gene. (C-H) GSEA enrichment plots showed correlated pathways of SF3A2 (ns represents no significance, * p < 0.05, ** p < 0.01, *** p < 0.001).

To elucidate SF3A2's biological functions in CRC, we determined genes showing a significant correlation with SF3A2, illustrated in the heatmap visualization (Figure 5A). Subsequently, we conducted GO and KEGG pathway analyses for these identified genes. GO annotation demonstrated that genes co-expressed with SF3A2 (correlation coefficient > 0.5) were predominantly associated with mRNA processing, RNA splicing, spliceosomal complex assembly, and transcription coregulator activity (Figure 5B). Additionally, GSEA evaluation indicated that SF3A2 expression exhibited positive associations with MYC targets, DNA replication, and Notch signaling pathway while showing negative correlations with inflammatory response, cytokine receptor interaction, and drug metabolism cytochrome P450. These observations suggest SF3A2's potential role in CRC progression (Figure 5C-H).

### SF3A2 is associated with suppressive CRC immune infiltration

**Figure 6:**
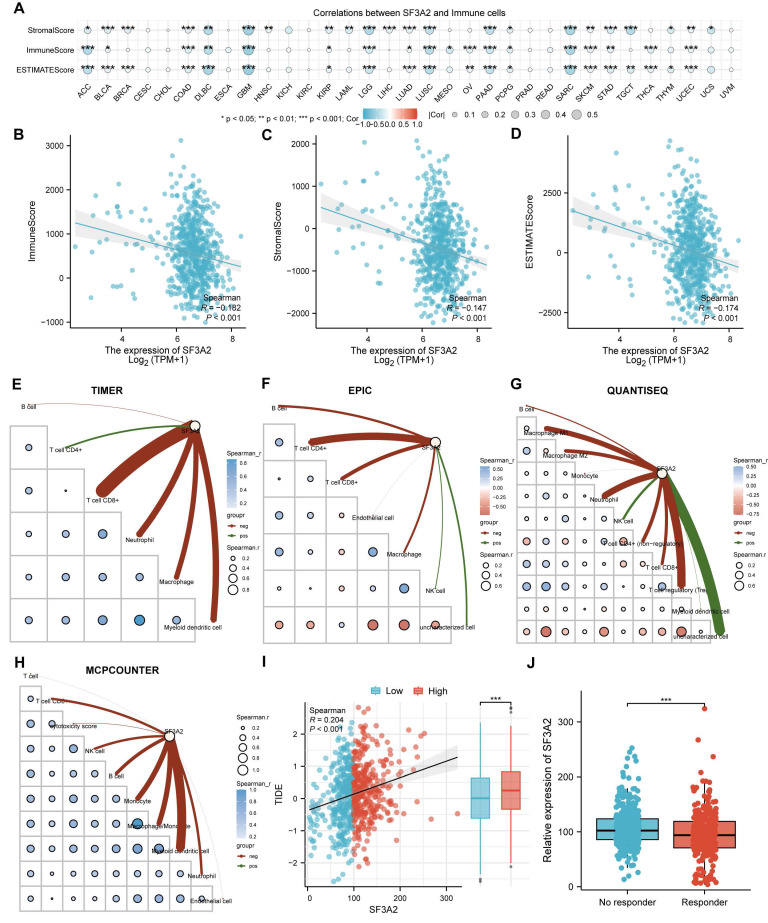
The correlation of SF3A2 expression with tumor microenvironment and immune infiltration level in CRC. A. The SF3A2 was negatively linked to stromal score, ESTIMATE score, and immune score in most tumor types from TCGA. B to D. The correlation between SF3A2 expression and the ESTIMATEScore, StromalScore, and ImmuneScore based on the ESTIMATE algorithm in CRC. E-H. The correlation of SF3A2 expression with immune cell infiltration in CRC acquired by TIMER, EPIC, QUANTISEQ and MCPCOUNTER. I. SF3A2 expression positively correlated with TIDE score in CRC. J. SF3A2 expression predicted immunotherapy response in CRC. (ns represents no significance, * p < 0.05, ** p < 0.01, *** p < 0.001).

Since the immune microenvironment markedly shapes tumor progression and offers insights into cancer immunotherapy strategies, we investigated the association between SF3A2 and the tumor immune landscape. To investigate SF3A2's interaction with the immune microenvironment across various cancers, we assessed the associations between SF3A2 levels and immune cell populations using ESTIMATE algorithms. The results indicated that SF3A2 was negatively linked to StromalScore, ESTIMATEScore, and ImmuneScore in most tumor types from TCGA (Figure 6A). Especially, SF3A2 was found to be significantly and negatively correlated with StromalScore, ESTIMATEScore, and ImmuneScore. (Figure 6B-D). Additionally, we employed TIMER, EPIC, QUANTISEQ and MCPCOUNTER computational approaches to assess tumor-infiltrating immune cell composition affecting the immune microenvironment status. The results showed that SF3A2 expression was negatively correlated with most immune cells infiltration levels including CD8^+^T cells, CD4^+^T cells, macrophages, and dendric cells (Figure 6E-H, Table 4). Subsequently, to evaluate the potential role of SF3A2 in tumor immune evasion, we analyzed its correlation with the TIDE score using transcriptomic data from TCGA CRC cohort. A significant positive correlation was observed between SF3A2 expression and TIDE score (Figure 6I). Consistent with this, patients with high SF3A2 expression exhibited significantly elevated TIDE scores compared to those with low SF3A2 expression (Figure 6I). We next stratified CRC patients into immunotherapy responder and non-responder groups based on established TIDE score thresholds. Strikingly, SF3A2 expression was significantly lower in the responder group compared to the non-responder group (Figure 6J). These findings indicated that high SF3A2 expression was associated with an immunosuppressive phenotype (high TIDE) and may serve as a novel biomarker for predicting resistance to immune checkpoint blockade in colorectal cancer.

## Discussion

Globally, CRC ranks among the leading contributors to cancer mortality. Despite considerable progress in therapeutic approaches, CRC remains a significant source of cancer-related deaths and requires a deeper understanding of molecular drivers 21, 22. Elucidating disease mechanisms at molecular and cellular levels remains essential for advancing clinical applications.

Proteins are the primary functional executors in cells, directly reflecting cellular physiology and function. Using proteomics technology, we performed a high-throughput analysis to identify key driving factors and potential mechanisms underlying CRC pathogenesis. Our investigation contrasted protein patterns between CRC specimens and neighboring healthy tissues through proteomics. We identified 290 differentially expressed proteins, comprising 131 elevated and 159 reduced proteins.

Further analysis through functional enrichment and pathway examination revealed abnormally elevated expression of RNA splicing-related proteins in cancerous tissues. Alternative pre-mRNA splicing enables the generation of multiple protein variants from individual genes^23^. Cancer cells often utilize this splicing flexibility to enhance the production of specific protein variants that promote tumor development. Research increasingly indicates that irregular mRNA splicing is fundamental to cancer initiation and advancement, influencing multiple critical pathways^24^. Many investigations have found links between the altered expression of splicing factors and tumor establishment, progression, and resistance to therapy^25^. In our research, 11 proteins involved in RNA splicing were found to be up-regulated in colorectal cancer, indicating an extensive alteration of dysregulation in the expression of splicing factors in CRC. Their associations with CRC patients' OS were further investigated by univariate Cox proportional hazards model. SF3A2 was considered as a critical mediator of tumor aggressiveness because of its association with poor patient prognosis.

SF3A2, an evolutionarily preserved element of U2 snRNP and a constituent of the vital mammalian splicing factor SF3a exhibits high conservation across species^26^. Nevertheless, research on SF3A2 remains limited, and its cancer-related functions are not well defined. Investigations indicated elevated SF3A2 expression in TNBC specimens, promoting disease advancement as demonstrated through various cellular assays. These observations aligned with our CRC findings, suggesting SF3A2's potential as a therapeutic target. Research examining SF3A2's tumor-related mechanisms remained sparse. Li et al demonstrated that SF3A2 enhanced TNBC progression by modulating makorin ring finger protein 1 (MKRN1) alternative splicing and boosting the dominant oncogenic variant, MKRN1-T1e. In this research, we further sought to predict the potential molecular mechanism of SF3A2 using bioinformatic analyses. It was revealed that SF3A2 was extensively involved in the selective splicing of RNA and transcription coregulation in CRC through the analysis of gene co-expression networks and gene ontology. Furthermore, the signaling pathways that might be associated with SF3A2 were analyzed via the GSEA. The MYC signaling pathway, DNA replication, and Notch signaling pathway were positively related to SF3A2, while the inflammatory response and cytokine-cytokine receptor interaction were negatively related to SF3A2. The MYC proto-oncogene family comprises three closely associated nuclear phosphoproteins: c-MYC, N-MYC, and L-MYC. Expression levels of the *c-MYC* oncogene exceed normal limits in approximately 70%-80% of colon adenocarcinoma cases^27^. DNA duplication is vital in cellular multiplication and enables unlimited proliferative capacity, a distinctive characteristic of cancer cells^28^. The Notch pathway serves essential functions in cellular differentiation, viability, multiplication, stem cell maintenance, growth, and tissue formation. Within CRC, disrupted Notch signaling affects various biological functions, including cell growth, invasiveness, cell cycle regulation, immune system avoidance, treatment resistance, and stem cell properties^29^. These observations helped explain the mechanisms through which SF3A2 drives CRC oncogenesis. While our bioinformatic analyses strongly associated SF3A2 with these key pathways, future research is essential to determine the principal SF3A2-mediated alternative splicing networks explore the specific downstream effectors that contribute to CRC tumorigenesis.

The composition and functional state of the TME, particularly the presence and activity of cytotoxic T lymphocytes (CTLs), are pivotal factors governing response to ICB. Tumors can be broadly categorized as “hot” (inflamed, T-cell-infiltrated), “excluded” (T-cells at the periphery but not penetrating the tumor core), or “cold” (immune-deserted, lacking T-cell infiltration). Cold tumors, characterized by minimal effector immune cell infiltration and an immunosuppressive milieu, represent a major challenge for immunotherapy. Understanding the molecular drivers that sculpt the TME towards an immune-desert phenotype is therefore paramount for developing strategies to overcome ICB resistance in CRC^30^, ^31^.

Notably, our analysis revealed an inverse correlation between SF3A2 levels and immune-related pathways, suggesting SF3A2's role in creating an immunosuppressive environment. Investigations have demonstrated the essential role of the CRC immune microenvironment in disease initiation and advancement. Additionally, the distribution of immune cells within the tumor microenvironment (TME) markedly shapes anti-cancer treatment outcomes^32^, ^33^. Our study revealed a significant correlation between elevated SF3A2 expression and an immunosuppressive tumor microenvironment (TME) in CRC. Using four independent computational algorithms (TIMER, EPIC, QUANTISEQ, and MCPCOUNTER), we consistently demonstrated that high SF3A2 expression was associated with markedly reduced infiltration levels of multiple anti-tumor immune cell subsets, including CD8^+^ T cells, dendritic cells, and M1 macrophages. This pattern collectively pointed to an “immune-desert” or “cold tumor” phenotype in SF3A2-high tumors, characterized by a paucity of effector immune cells within the TME. Such a landscape is often linked to immune evasion and poor responses to immunotherapies. Further supporting the dysfunctional immune state, our TIDE (Tumor Immune Dysfunction and Exclusion) analysis revealed significantly higher TIDE scores in patients with high SF3A2 expression. The TIDE score is a validated computational framework that integrates signatures of T-cell dysfunction and exclusion to predict immune checkpoint blockade (ICB) resistance. The elevated TIDE score in the SF3A2-high group strongly suggested the presence of mechanisms that either impair T-cell function or prevent T-cell recruitment into the tumor core. Crucially, when utilizing TIDE to predict clinical response to ICB therapy, patients with high SF3A2 expression exhibited a significantly lower predicted response rate. This computational prediction aligned with the observed “cold” immune phenotype and underscored the potential of SF3A2 as a novel biomarker for intrinsic resistance to immunotherapy in CRC.

The association of SF3A2, a core component of the spliceosome's SF3a complex, with cancer progression and an immunosuppressive TME is a novel and intriguing finding. While SF3A2's primary role is in pre-mRNA splicing, its dysregulation in cancer may have profound downstream consequences. We hypothesized that SF3A2 overexpression could potentially drive tumor progression and immune evasion through several plausible mechanisms: (1) Oncogenic splicing programs: SF3A2 might promote the expression of pro-tumorigenic splice variants of genes directly involved in tumor progression and immune inhibition. (2) Altered antigen presentation: Dysregulated splicing of genes involved in antigen processing and presentation could impair tumor antigen recognition by T cells. (3) Immunomodulatory factor splicing: Aberrant splicing of cytokines, chemokines, or their receptors (e.g., CXCL9, CXCL10, IFN-γ pathway genes) could disrupt critical immune cell recruitment and activation signals. Indeed, further research is essential to explore SF3A2's biological roles and its core regulatory mechanisms during CRC initiation and progression. Firstly, given SF3A2's function as a splicing factor, it is necessary to determine the principal SF3A2-mediated AS networks that contribute to CRC tumorigenesis. Secondly, the role of SF3A2 in inducing a suppressive immune environment and influencing the effect of immunotherapy needs to be verified in vivo and its predictive value for immunotherapy should be validated in prospective cohorts of ICB-treated CRC patients. Finally, targeting SF3A2 or its downstream splicing events might represent a promising avenue for overcoming immunotherapy resistance in this molecularly defined subset of CRC.

While our study established SF3A2's role in CRC progression and immunosuppression, we acknowledged its limitations and identified clear paths for future validation. A key priority is to verify the universality and clinical robustness of SF3A2 as a biomarker in large, multi-center, independent external cohorts. Furthermore, future studies should directly compare the predictive performance of SF3A2 against established and emerging immune biomarkers, such as PD-L1 expression, tumor mutational burden (TMB), and MSI status, to determine its independent and complementary value in clinical decision-making. Investigating the interplay between SF3A2-driven splicing programs and these canonical immune pathways will provide a more comprehensive understanding of the mechanisms shaping the immunosuppressive TME in CRC. Addressing these questions will be crucial for translating our findings into clinically actionable strategies.

In conclusion, our investigation demonstrated elevated SF3A2 expression within CRC tissue samples, correlating with unfavorable outcomes in CRC patients. Our findings emphasized SF3A2's significant role in promoting CRC advancement and suppressive immune microenvironment. Consequently, this investigation indicated SF3A2's potential utility as a therapeutic intervention point and prognostic indicator for CRC.

## Supplementary Material

Supplementary tables.

## Figures and Tables

**Table 1:** Univariate and multivariate Cox proportional hazards model of factors affecting the OS of CRC patients in the TCGA database

Characteristics	Total(N)	Univariate analysis			Multivariate analysis	
		Hazard ratio (95% CI)	P value		Hazard ratio (95% CI)	P value
Pathologic T stage	556					
T1&T2	115	Reference			Reference	
T3&T4	441	3.404 (1.578 - 7.339)	**0.002**		2.246 (1.018 - 4.956)	**0.045**
Pathologic N stage	556					
N0	327	Reference			Reference	
N1&N2	229	2.789 (1.888 - 4.120)	**< 0.001**		0.507 (0.193 - 1.330)	0.167
Pathologic M stage	556					
M0	468	Reference			Reference	
M1	88	4.086 (2.746 - 6.080)	**< 0.001**		2.496 (1.542 - 4.041)	**< 0.001**
Pathologic stage	556					
Stage I&Stage II	317	Reference			Reference	
Stage III&Stage IV	239	3.279 (2.191 - 4.906)	**< 0.001**		4.253 (1.448 - 12.487)	**0.008**
Gender	556					
Female	264	Reference				
Male	292	0.941 (0.646 - 1.372)	0.753			
Age	556					
<= 65	234	Reference			Reference	
> 65	322	2.246 (1.454 - 3.471)	**< 0.001**		2.816 (1.811 - 4.377)	**< 0.001**
SF3A2	556	1.413 (1.022 - 1.955)	**0.037**		1.397 (1.018 - 1.916)	**0.038**

**Table 2:** Univariate and multivariate Cox proportional hazards model of factors affecting the DSS of CRC patients in the TCGA database

Characteristics	Total(N)	Univariate analysis			Multivariate analysis	
		Hazard ratio (95% CI)	P value		Hazard ratio (95% CI)	P value
Pathologic T stage	535					
T1&T2	113	Reference			Reference	
T3&T4	422	5.765 (1.810 - 18.362)	**0.003**		3.192 (0.967 - 10.539)	0.057
Pathologic N stage	535					
N0	318	Reference			Reference	
N1&N2	217	4.230 (2.492 - 7.179)	**< 0.001**		0.556 (0.211 - 1.464)	0.235
Pathologic M stage	535					
M0	449	Reference			Reference	
M1	86	7.776 (4.816 - 12.555)	**< 0.001**		4.094 (2.277 - 7.361)	**< 0.001**
Pathologic stage	535					
Stage I&Stage II	308	Reference			Reference	
Stage III&Stage IV	227	6.001 (3.335 - 10.800)	**< 0.001**		4.679 (1.409 - 15.531)	**0.012**
Gender	535					
Female	254	Reference				
Male	281	1.039 (0.646 - 1.669)	0.876			
Age	535					
<= 65	232	Reference			Reference	
> 65	303	1.581 (0.958 - 2.609)	0.073		2.169 (1.303 - 3.612)	**0.003**
SF3A2	535	1.539 (1.021 - 2.319)	**0.040**		1.527 (1.018 - 2.290)	**0.041**

**Table 3:** Univariate and multivariate Cox proportional hazards model of factors affecting the PFI of CRC patients in the TCGA database

Characteristics	Total(N)	Univariate analysis			Multivariate analysis	
		Hazard ratio (95% CI)	P value		Hazard ratio (95% CI)	P value
Pathologic T stage	556					
T1&T2	115	Reference			Reference	
T3&T4	441	2.791 (1.578 - 4.936)	**< 0.001**		1.857 (1.025 - 3.365)	**0.041**
Pathologic N stage	556					
N0	327	Reference			Reference	
N1&N2	229	2.401 (1.729 - 3.335)	**< 0.001**		0.953 (0.406 - 2.234)	0.911
Pathologic M stage	556					
M0	468	Reference			Reference	
M1	88	5.820 (4.108 - 8.243)	**< 0.001**		4.441 (2.849 - 6.925)	**< 0.001**
Pathologic stage	556					
Stage I&Stage II	317	Reference			Reference	
Stage III&Stage IV	239	2.715 (1.944 - 3.791)	**< 0.001**		1.355 (0.524 - 3.504)	0.530
Gender	556					
Female	264	Reference				
Male	292	1.109 (0.800 - 1.537)	0.537			
Age	556					
<= 65	234	Reference				
> 65	322	1.094 (0.785 - 1.525)	0.597			
SF3A2	556	1.138 (0.902 - 1.436)	0.274			

**Table 4:** Correlation of SF3A2 expression with immune infiltration in CRC.

Category	Immune cell	Relevance	P value
TIMER	T cell CD8^+^	-0.32086	2.60E-16
	Neutrophil	-0.21325	8.91E-08
	Macrophage	-0.18432	3.84E-06
	Myeloid dendritic cell	-0.1751	1.20E-05
	B cell	-0.03424	0.394691
	T cell CD4^+^	0.079723	0.047228
EPIC	T cell CD4^+^	-0.16443	3.98E-05
	T cell CD8^+^	-0.11741	0.003432
	B cell	-0.09136	0.022927
	Macrophage	-0.08496	0.034453
	Endothelial cell	-0.0093	0.817133
	NK cell	0.056482	0.160075
	uncharacterized cell	0.07924	0.048609
QUANTISEQ	Neutrophil	-0.17465	1.26E-05
	T cell regulatory (Tregs)	-0.17459	1.23E-05
	Macrophage M1	-0.15311	0.000132
	Macrophage M2	-0.11609	0.003797
	T cell CD4^+^ (non-regulatory)	-0.11317	0.004783
	T cell CD8^+^	-0.10571	0.008433
	B cell	-0.06322	0.115838
	Monocyte	-0.01426	0.723055
	Myeloid dendritic cell	0.026346	0.512595
	NK cell	0.09135	0.02292
	uncharacterized cell	0.244025	8.42E-10
MCPCOUNTER	Myeloid dendritic cell	-0.21569	6.30E-08
	Monocyte	-0.15598	9.81E-05
	Macrophage/Monocyte	-0.15598	9.81E-05
	B cell	-0.11108	0.005648
	Neutrophil	-0.0952	0.01777
	NK cell	-0.09251	0.021258
	T cell CD8^+^	-0.09251	0.02124
	cytotoxicity score	-0.03847	0.338838
	Endothelial cell	-0.01514	0.706743
	T cell	0.016752	0.677094
